# Coronavirus: just imagine…

**DOI:** 10.1186/s13054-020-2824-8

**Published:** 2020-03-16

**Authors:** Jean-Louis Vincent, Arthur S. Slutsky

**Affiliations:** 1 Department of Intensive Care, Erasme University Hospital, Université Libre de Bruxelles, Route de Lennik 808, 1070 Brussels, Belgium; 2 Keenan Research Center for Biomedical Science at the Li Ka Shing Knowledge Institute, St. Michael’s Hospital, Toronto, Canada; 3 Departments of Medicine, Surgery, and Biomedical Engineering, University of Toronto, Toronto, Canada

Just imagine this possible scenario: it is summer 2020 and our intensive care unit (ICU) is full of patients with COVID-19 and severe respiratory failure as a result of the newly described coronavirus, SARS-CoV-2 [1–3]. We have decreased virtually all the other activities in the hospital, so that there are not many critically ill patients with other problems. We have cancelled all non-urgent (and in fact some urgent) surgical cases, including cardiac surgery and neurosurgery cases that usually come through our Department.

The pandemic is now out of control, despite the advances in global health that have taken place since severe acute respiratory syndrome (SARS) in 2003 and Middle East respiratory syndrome (MERS) in 2012. The trigger event that led to this rapid, overwhelming spread of the infection seems to have been the admission of an infected Chinese worker to a hospital somewhere in Central Africa, where healthcare facilities are more limited and testing not widely available. Control measures while waiting for results were apparently insufficient and the virus spread rapidly among the local population and then elsewhere. With the huge number of cases infected, quarantine is no longer possible or practicable and has been virtually abandoned. The total number of cases worldwide now exceeds 1 million in 59 countries, with close to 50,000 deaths (about 5%) attributed to the infection. One would assume that these were primarily in elderly patients with multiple co-morbidities, but this is not true: many relatively young and previously healthy individuals have also died. The concern we all have is that this may be the *new normal*.

Our ICU is overflowing with patients. Yet there are more waiting in the emergency room, and many patients on the regular wards are getting worse. We cannot transfer patients to other institutions, because they have been affected to a similar extent. Currently, we have 8 patients on extracorporeal membrane oxygenation (ECMO) and desperately need more machines. The cardiac surgery program has essentially stopped so that we can use their ECMO circuits, and we do not know where to get more as the manufacturers have none available. They have increased factory production, but we do not know when the next machine will be ready. Worse, even if the units arrive, we do not think we will have enough personnel to run them.

Should we simply let these extremely sick patients die so that the less sick can be treated in a more effective manner? At least one of our patients on ECMO will definitely not make it. If it were up to us, we would discontinue our efforts for that patient, but the patient’s family is very much against any withdrawal of life support. We will try again to convince them that their relative will not survive and that further treatment is of no benefit, but we are not confident that they will change their minds. In the midst of all of this, our hospital director insists we admit his brother with severe pneumonia….

Our entire ICU team is burned out. Some of them are also infected; for others, we are not sure. Mark called yesterday to say he was not feeling well, but it is unclear if this is more psychological than somatic: he found the strain of wearing the protective gear, with the associated heat and claustrophobic feeling, really difficult. He was concerned about the complex process of simply going to the bathroom, not to mention the underlying fear we all have of catching the virus ourselves. Dominique did not come in today, in part because no one was willing to take care of her small children; everyone is afraid to be close to healthcare workers and their families, perhaps with some justification.

We have tried to get support from other sectors in the hospital, like the operating room where there are very few operations taking place or the general medical and surgical floors. But, these nurses and doctors are not trained in critical care management and are a bit lost when faced with a respirator and even more so with ECMO. To be honest, we are not sure we will be able to keep all the protective measures in place, because the situation is out of control. We do not know what will happen.

Is this really just a bad dream? Maybe. We can only hope it does not come true…..

*Footnote:*

“The authors wish to warn readers that the scenario portrayed is a fictional account, and not a prediction of the future.”

## Data Availability

Not applicable

## Abbreviations

ICU: Intensive care unit
ECMO: Extracorporeal membrane oxygenation
MERS: Middle East respiratory syndrome
SARS: Severe acute respiratory syndrome
